# Acne Severity and Sleep Quality in Adults

**DOI:** 10.3390/clockssleep1040039

**Published:** 2019-12-06

**Authors:** Kory P. Schrom, Sayeeda Ahsanuddin, Michelle Baechtold, Raghav Tripathi, Amy Ramser, Elma Baron

**Affiliations:** 1 Department of Dermatology, University Hospitals Cleveland Medical Center, Cleveland, OH 44106, USA; Sayeeda.Ahsanuddin2@UHhospitals.org (S.A.); mfilanovsky@gmail.com (M.B.); rxt177@case.edu (R.T.); edb4@case.edu (E.B.); 2 Department of Dermatology, Case Western Reserve University School of Medicine, Cleveland, OH 44106, USA; 3 Department of Dermatology, Riverside Methodist Hospital, Columbus, OH 43214, USA; bethramser@gmail.com; 4 Department of Dermatology, Louis Stokes Cleveland Veterans Affairs Medical Center, Cleveland, OH 44106, USA

**Keywords:** sleep, circadian rhythm, acne vulgaris

## Abstract

Poor sleep quality is extremely prevalent, with about one third of adults in the USA obtaining less than the recommended amount of sleep. In addition, poor sleep quality has been linked to an increased risk of many conditions, including diabetes, hypertension, psychiatric conditions, and overall all-cause mortality. Research has shown that sleep disturbance does impact skin disease, although many details of this relationship are still unclear. The goal of this study is to determine if there is a relationship between acne severity and sleep quality in adults. Forty subjects with acne were recruited from dermatology clinics in Cleveland, OH, to participate in this study. Acne severity was assessed using the Global Acne Grading Scale (GAGS). To assess sleep quality, subjects completed the Pittsburgh Sleep Quality Index (PSQI) and completed a seven-day sleep journal. Subjects also completed the Dermatology Life Quality Index (DLQI), the Patient Health Questionnaire-2 (PHQ-2), and provided information about current and past acne treatments as well as their opinion regarding their own acne severity and exacerbating factors. Our findings support the hypothesis that there is a potential relationship between sleep quality and acne.

## 1. Introduction

Sleep is critical for normal human biological and physiological functioning, and it is partly regulated by the suprachiasmatic nuclei located in the anterior hypothalamus. These nuclei function as a central “clock” which coordinates biological processes that occur at recurring intervals over an approximately 24-hour period of time known as the circadian rhythm [1]. This central “clock” regulates normal functioning by coordinating with peripheral “clocks” that are expressed in cells throughout the body, including the skin [2,3,4,5,6]. 

In the skin, temperature variations, pH, barrier function, and transepidermal water loss have been shown to vary depending on the time of day [7]. Previous work by our group has demonstrated that poor sleep quality is associated with increases in signs of intrinsic skin aging, poor skin barrier repair, and poor perceptions of appearance [8]. However, the mechanisms behind our findings remain unclear and may be the result of increased oxidative stress secondary to poor sleep [9,10]. Sleep and its impact on inflammatory skin conditions has also been alluded to in psoriatic animal models wherein acute sleep deprivation was linked to worsening psoriatic inflammation; however, data evaluating sleep in the context of human inflammatory skin disorders, including acne vulgaris (acne), is limited [11]. 

Acne is a chronic inflammatory dermatosis affecting the pilosebaceous unit that is characterized by open and closed comedones as well as inflammatory papules, pustules, and cysts. It affects nearly 50 million individuals in the United States. Groups specifically affected include 85% of teenagers and 12% of adult females. Its pathogenesis involves follicular hyperkeratinization, inflammation, in part generated by *Cutibacterium acnes* (formerly *Proprionibacterium acnes)* and hormonal alterations [12]. In a French study, fatigue upon waking and acne were shown to be strongly correlated [13]. Prior studies have also suggested a bi-directional relationship between sleep and acne [14,15]. In this study, we attempted to further elucidate the connection between sleep and acne. 

## 2. Results

### 2.1. Demographics

Forty subjects were enrolled in our study, with the majority being female (82.5%) and white (57.5%). Thirty subjects (75%) were actively being treated for acne at the time of the study, and only three (7.5%) were previously diagnosed with a sleep disorder. As determined by the Global Acne Grading Scale (GAGS), acne severity of our cohort ranged from mild to severe, with the majority of subjects being classified as mild (*n* = 21), followed by moderate (*n* = 17); two were classified as severe. Thirty subjects scored a 5 or greater on the Pittsburgh Sleep Quality Index (PSQI) indicating poor sleep quality. Demographic and acne/sleep characteristics of the population are detailed in Table 1.

### 2.2. Correlational Analysis

Univariate analysis using Pearson’s correlation coefficient (*r*) showed several statistically significant findings. Self-reported acne severity was directly correlated with Dermatology Life Quality Index (DLQI) (*r* = 0.44677; *p* = 0.0039) and Patient Health Questionnaire-2 (PHQ-2) (*r* = 0.33512, *p* = 0.0345), respectively. Other directly correlated parameters were PSQI and DLQI (*r* = 0.48392, *p* = 0.0016), PSQI and PHQ-2 (*r* = 0.52008, *p* = 0.0006), as well as DLQI and PHQ-2 (*r* = 0.49736, *p* = 0.0011). Inversely correlated parameters included PSQI and average subjective sleep score (*r* = −0.43494 *p* = 0.0337) and DLQI and average subjective sleep score (*r* = −0.64880, *p* = 0.0006). 

Self-reported acne severity and GAGS were not correlated (*r* = 0.21182, *p* = 0.1895) and neither were self-reported acne severity and PSQI (*r* = 0.19349, *p* = 0.2316). There were no correlations between PSQI and GAGS (*r* = −0.14822, *p* = 0.3614), GAGS and DLQI (*r* = −0.06392, *p* = 0.6952), GAGS and PHQ-2 (*r* = −0.00332, *p* = 0.9838), PHQ-2 and average subjective sleep score (*r* = −0.33288, *p* = 0.1120), as well as GAGS and average subjective sleep score (*r* = −0.14667, *p* = 0.4940)

### 2.3. Multiple Least Squares Regression Models

Controlling for DLQI and PHQ-2, average subjective sleep score is dependent on GAGS (*p* = 0.0306, parameter estimate= −0.09003). Controlling for DLQI and PHQ2, PSQI is not dependent on GAGS (*p* = 0.2271). Controlling for PSQI, self-reported acne severity is not dependent on GAGS (*p* = 0.1282). Controlling for PSQI and PHQ-2, self-reported acne severity is not dependent on GAGS (*p* = 0.2159). Controlling for PSQI, PHQ-2, and DLQI, self-reported acne severity is not dependent on GAGS (*p* = 0.1486). 

Sample size (*n*) was equal to 24, *p* = 0.0011 for the model and *r*^2^ = 0.5448. Sixteen of the rows in the model had missing rows and could not be used. For each individual parameter, *p* = 0.0306 for GAGS (parameter estimate = −0.09003), *p* = 0.0003 for DLQI (parameter estimate = −0.26909), and *p* = 0.3772 for PHQ2 (parameter estimate = 0.15963).

## 3. Discussion

Our study showed that, on univariate analysis, self-reported acne severity scores are directly correlated with poorer quality of life and depressive symptoms (DLQI: *r* = 0.44677, *p* = 0.0039; PHQ-2: *r* = 0.33512, *p* = 0.0345). On multivariate analysis, when controlling for DLQI and PHQ-2, the average subjective sleep score decreased (i.e., worsened) as the objective acne severity score (GAGS) increased and vice versa (*p* = 0.0306, parameter estimate = −0.09003). In 2015, a French study demonstrated that there is a strong positive correlation between acne and fatigue upon waking (i.e., poor sleep quality) even when adjusting for age (*p* < 0.0001). It also demonstrated stressed patients have greater fatigue upon waking (*p* < 0.0001) and are more likely to have acne (OR = 1.975; *p* < 0.0001) [13]. Although fatigue may be a symptom of depression and patients whose quality of life has been more severely impacted by acne are more stressed, our research demonstrates that, when controlling for depressive symptoms and quality of life impact, subjectively worse sleep quality is associated with objectively worse acne [13,16]. This suggests that there may be an influence of acne severity on subjective sleep quality and vice versa, confirming prior findings of the impact acne has on quality of life and mental health [13,16,17,18].

Our data did not establish a correlation between the PSQI and either subjective (*r* = 0.19349, *p* = 0.2316) or objective (GAGS; *r* = −0.14822, *p* = 0.3614) scores of acne severity. There was also no correlation (*p* = 0.2271) between the PSQI and GAGS even when controlling for the DLQI and PHQ-2 like there was for average subjective sleep scores and GAGS. In addition, it is interesting to note that there was no correlation (*r* = 0.21182, *p* = 0.1895) between self-reported acne severity and GAGS, even when controlling for the PSQI, PHQ-2, and DLQI (*p* = 0.1486). The GAGS in our study may have been more congruent with our patient’s self-reported acne severity had our patients been more severe as determined by the GAGS. This seems likely given that prior research has shown that a patient’s perception of their acne is directly correlated to the impact it has on their quality of life, and objective severity does not always correlate with the impact of the disease experienced by the patient [19,20]. Future research should include subjects with greater acne severity given the impact acne severity has on quality of life, stress, and mental health [13,16,17,18].

Further limitations of our study were its smaller sample size and the lack of a physiological measure of sleep quality (e.g., polysomnography). A power analysis was not performed for our study. Our sample size was determined based on review of similar research and the assumption that our sample would be normally distributed. Informed by our experience and findings, future studies with larger cohorts are necessary to both confirm our findings and to ascertain whether or not a type II error occurred for our non-significant findings due to a lack of power. Additionally, although the PSQI generates a standardized score, it still is based on self-reporting and may be less reflective of actual sleep disturbances [21].

Despite these limitations, our data and the data of others does suggest a complex relationship between sleep and acne. The relationship between acne and sleep quality are likely the consequence of a dynamic interplay between psychiatric and pathophysiologic factors given the correlation among DLQI, PHQ-2, and sleep quality. This is further supported by the correlation between sleep quality and objective acne severity rather than subjective severity demonstrated in this analysis. Insufficient sleep duration has been linked to numerous inflammatory systemic diseases, including diabetes, hypertension, obesity, psychiatric diseases, and increased all-cause mortality [17]. Several mechanisms have been postulated to explain these relationships. In one study, using in situ hybridization, facial skin biopsies from patients with acne were found to have higher expression of corticotropin-releasing hormone receptors in acne-involved sebaceous glands when compared with normal and uninvolved skin. These findings imply a role in stress-induced, sleep-related development and exacerbation of acne [22]. 

There are also numerous studies exploring the inter-relationship between the internal circadian clock and the commensal microbiome as it relates to host immune function as well as digestive and metabolic functions [23,24]. Studies have demonstrated greater metagenomic diversity of *C. acnes* in patients with acne vulgaris versus their healthy skin counterparts, and it would be interesting to investigate the influence of circadian function specifically on cutaneous *C. acne* populations [25]. The relationship among sleep disturbances, mental health, and the skin microbiome in patients with acne is an area for further investigation. 

## 4. Materials and Methods 

### 4.1. Study Design

This was a single-center, non-interventional research study conducted in the Department of Dermatology at University Hospitals Cleveland Medical Center in Cleveland, OH. The study received approval from the University Hospitals Institutional Review Board (IRB). There was no amendment to the trial protocol after it was initiated.

### 4.2. Recruitment and Study Population

Patients were identified and invited to join the study through dermatology clinics, local advertisements, hospital-wide recruitment events, and our department’s own research patient database. Inclusion criteria required subjects to be 18 years of age or older, have acne vulgaris, be in general good health, able to provide informed consent, and able to comply with all study activities. Subjects were considered to be in “general good health” if life-threatening acute medical conditions (e.g., active infection, cancer, etc.) were absent, and chronic medical conditions (e.g., diabetes, hypertension, etc.) were well controlled based on investigator assessment. Women who were pregnant or actively nursing were excluded. Active treatment for acne vulgaris was neither required nor prohibited. 

### 4.3. Interventions

All subjects underwent a full medical history including an acne-specific medical history. This medical history review included review of current and previous acne therapies. For female patients, a brief menstrual history was also recorded. Acne severity was assessed at a single point in time using the Global Acne Grading System (GAGS). Subjects were asked to rate the severity of their acne on the day of enrollment on a scale of 0–10 (with 0 being completely clear, and 10 being the worst acne the subject had experienced in the past six months). Depressed mood was assessed using the Patient Health Questionnaire 2 (PHQ-2), the impact on quality of life caused by acne was assessed with the Dermatology Life Quality Index (DLQI), and the Pittsburgh Sleep Quality Index (PSQI) was used to assess sleep quality over the past month. Subjects were also given the option to complete a sleep journal which they completed daily for seven consecutive days following their in-person study visit. The journal included a subjective sleep quality rating for each night (scale of 1 to 10, with 1 being very poor and 10 being excellent). Their sleep journal was subsequently returned after its completion.

### 4.4. Outcome Measures

The two primary outcome measures in this study are the relationship between acne severity (i.e., GAGS and self-reported) and sleep quality (PSQI and self-reported). Secondary outcomes measured included the correlation between objective or subject acne severity and depressed mood (PHQ-2) as well as the impact on quality of life (DLQI).

### 4.5. Statistical Analysis

After descriptive analysis of sociodemographic, acne, and sleep characteristics, Pearson’s correlational coefficient was utilized for univariate comparison of linear variables in this study (including PSQI, DLQI, PHQ2, GAGS, and self-reported acne severity). Five multivariable linear regression models were then created to evaluate the relationships between continuous variables using the multiple least squares regression technique. In all models, the predictor variable was GAGS. In the first model, DLQI and PHQ2 were covariates and average sleep score was the outcome variable. In the second model, DLQI and PHQ2 were covariates and PSQI was the outcome variable. For models 3–5, self-reported acne severity was the outcome variable and PSQI, PHQ-2, and DLQI were added in succession as predictor variables. Subjects with partially missing data were excluded from analysis as appropriate. *P* < 0.05 was considered significant for all statistical tests. SAS 9.4 (Cary, NC, USA) was utilized for all statistical analyses.

## 5. Conclusions

The correlation of acne severity with sleep quality is likely the consequence of a dynamic interplay of both psychiatric and pathophysiologic mechanisms. This analysis is an initial attempt to further elucidate the connection, but further research and analysis is required. Informed from this analysis, greater clarity on this subject matter will likely be gleaned from analyses with larger cohorts who have more severe acne and whose sleep quality is assessed on a more physiologic level (e.g. polysomnography). 

## Figures and Tables

**Table 1:** Sociodemographic and outcome variables of the study population.

Patient Demographics	Average (SD)
Age	31.18 (11.32)
Sex (*n*)	
Female	33
Male	7
Race (*n*)	
White	23
Black	12
Asian	4
Acne severity (*n*)	
Mild	21
Moderate	17
Severe	2
GAGS	17 (6.82)
Self-Rated Acne Score	4.73 (2.44)
DLQI	4.63 (5.50)
PHQ-2	1.10 (1.68)
PSQI	7.00 (3.71)
Age of Acne Onset (y)	14.13 (5.53)
Self-rated Sleep Score	6.96 (1.60)

## Abbreviations

DLQI	Dermatology Life Quality Index
PSQI	Pittsburgh Sleep Quality Index
PHQ-2	Patient Health Questionnaire-2
GAGS	Global acne grading scale
